# Distortional effects of separate accounting and formula apportionment on factor allocation

**DOI:** 10.1007/s11573-022-01133-5

**Published:** 2023-01-06

**Authors:** Regina Ortmann, Erich Pummerer

**Affiliations:** 1 Faculty of Business Administration and Economics, Universität Paderborn, Paderborn, Germany; 2 Department of Accounting, Auditing and Taxation, Universität Innsbruck, Innsbruck, Austria

**Keywords:** Factor allocation, Formula apportionment, Investment decision, Separate accounting, Transfer pricing, H21, H25, H26

## Abstract

We examine distortions caused by tax base allocation systems–separate accounting (SA) or formula apportionment (FA)–with respect to the allocation of assets and workforce within multinational entities (MNEs). The effects of both systems are intensively debated by EU Member States as they are striving to implement a European tax system. Its introduction would lead to a switch from SA to FA. Moreover, Pillar One of the recent global tax reform includes a mix of both tax base allocation systems. We find that, against the claims of the EU, FA does not necessarily create lower distortions of the factor allocation. Decisive for that assessment is the level of profit shifting under SA. Our results indicate that, in tendency, the factor allocation is more severely distorted by FA when the profit shifting possibilities were rather low under SA. In contrast to former studies, we highlight the importance of analyzing the status quo under the recently applied system (SA) in order to be able to assess the consequences of a switch from SA to FA. Our results are interesting for policy-makers as they help anticipating reactions of MNEs to a change in the applied tax base allocation system and for companies as a basis for future tax planning.

**Supplementary Information:**

The online version contains supplementary material available at 10.1007/s11573-022-01133-5.

## Introduction

We examine how the two major tax base allocation systems splitting profits of multinational entities (MNEs) between countries cause distortions of MNEs’ factor allocation. Separate accounting (SA) as one system is currently used in Europe and in most countries around the world. Under SA, each entity of an MNE is treated distinctly and has to calculate its tax liability separately according to national tax laws. In case of intra-group trade, transfer prices are applied to determine the allocation of profits between different jurisdictions. Under formula apportionment (FA), the uniformly determined profits and losses of the entities are consolidated on the group level and are subsequently allocated to the entities according to a specific apportionment formula. This formula is designed to capture the economic share contributed by each entity to the MNE’s profits. FA is already well-known as it is in use for corporate taxation, e.g., on the subnational level in the US, Canada and Germany. As both systems are based on a fundamentally different mechanism for determining the tax base per entity, they offer different incentives with respect to the favorable country of investment and with respect to profit shifting. Whereas under FA, profit shifting is realized via real activity shifting, under SA, book-profit shifting by transfer pricing is possible and, additionally, real activity shifting plays a role as investments in asset and workforce create tax-deductible expenses. Other profit shifting channels like internal debt shifting exist under SA. However, we focus here solely on transfer pricing.

The effects of both tax base allocation systems have been intensively debated, especially in Europe, against the background of the OECD BEPS project (OECD 2013). The goal of the OECD BEPS project is to tackle tax avoidance of MNEs by closing loops and mismatches in national tax codes. Action points 8, 9, 10 and 13 of the OECD BEPS project address profit shifting via transfer prices and propose more suitable and stricter regulations. Against this background, the proposal of a common harmonized tax system for Europe based on FA—the so-called common consolidated corporate tax base (CCCTB) —has been re-discussed by European Member States. In 2011, the European Commission proposed a Council Directive on a CCCTB (see European Commission 2011). According to this proposal, MNEs need to apply only one tax code to determine their tax base in European countries. The consolidated group tax base is allocated to each entity according to an apportionment formula based on assets, labor and sales. However, this proposal proved too ambitious for Member States to agree in one go (see European Commission 2016a). The European Commission regards the CCCTB as a more holistic solution for all kinds of BEPS issues, especially with respect to transfer pricing. Many Member States hold objections against the elements of consolidation and formula apportionment, which in fact constitutes a sensitive infringement of their tax sovereignty. Nonetheless, there was still strong demand for the benefits that the CCCTB could offer to Member States and MNEs in the EU (see European Commission 2016a). Therefore, the Commission re-launched it in 2016 through a more manageable two-step process. In the first step, only the common base should be implemented. Then, in the next step, the more critical elements consolidation and formula apportionment should be introduced. As these two elements are the issue of greatest concern for the Member States, research in this field is needed to be better able to anticipate the resulting economic and tax effects.

Moreover, the application of a mix of both tax base allocation systems is a hot worldwide topic right now as under Pillar One of the global tax reform some taxing rights will be re-allocated over MNEs from their home countries to the market countries by a FA-like procedure based on sales. Overall, multinational profits are still determined in line with the SA approach based on transfer pricing. However, MNEs with global sales above EUR 20 billion and profitability above 10% will be covered by the new rules, with 25% of profit above the 10% threshold to be re-allocated to market jurisdictions via FA (see OECD 2021). Overall, only a few very big global players will be affected by Pillar One: According to Devereux and Simmler (2021) it will be 78 of the world’s 500 largest companies.

For being able to assess the effects arising from Pillar One of the global tax reform it is important to first understand the resulting effects under both systems separately. The effects of which tax base allocation system dominate the respective other depends on the share of overall profits that is taxed according to SA or FA.

In general, a basic prerequisite for a good tax system is neutrality. Economic entities ought to be taxed in such a way that the scarce resources of an economy are allocated in a welfare-enhancing manner. Thus, the tax system ought to be designed in such a way that it does not affect free market conditions. Consequently, economic entities’ decisions should not be affected and investment decisions should not be distorted by corporate taxation. Against this background, we investigate the extent to which SA and FA are capable of ensuring such neutrality with respect to locational investment decisions and identify conditions (e.g. the interplay of tax rates differentials, of the mix of input factors necessary for producing the final product and of costs for adjusting the MNE’s company structure for tax planning purposes) under which one or the other system causes more severe distortions. Thus, our contribution consists in identifying the conditions and circumstances under which SA or FA leads to more severs distortions of entrepreneurial decisions.

We develop an analytical model to address our research question, namely under which conditions which tax base allocation system causes more severe distortions to MNE’s factor allocation. We apply a two-country, two-entity setting in which the MNE is able to allocate a given total investment in asset and workforce to each entity. We base our analysis on a pre-tax optimum. We explain, why deviating from this pre-tax optimum—in our study due to tax reasons—is costly. We justify a quadratic cost function for non-optimality by referring to a Cobb–Douglas production function.

Taking non-optimality cost into account, we determine optimal after-tax allocation of resources under both tax allocation systems. The optimal after-tax allocation of resources is characterized by the highest after-tax cash flows under each system. The deviation of the after-tax optimum from the pre-tax optimum determines the level of distortion caused by each system. By comparing these differences, we can derive conclusions about the distortive power of each system—the greater the difference, the greater the distortive power. In our business-perspective-model, the MNE faces a trade-off between choosing a tax-optimal allocation of investment funds and an optimal allocation from a pre-tax perspective in order to minimize non-optimality costs.

Whereas investment decisions in connection with profit shifting behavior of MNEs have intensively been investigated under SA, there is little research on these aspects under FA. The effects of FA have so far mainly been investigated from a public finance perspective. There is only scarce literature from a business perspective. We are the first to identify the conditions that determine the distortional power inherent in both tax allocation systems with respect to investment decisions.

First, we find that, in contrast to claims of the European Commission, a shift from SA to FA does not necessarily result in less severe distortions. Second, in line with the results of Martini et al. (2012), yet in contrast to some commonly held views,^1^ we find that the application of transfer pricing under SA has real economic effects. Moreover, in contrast to the often-stated insensitivity of FA toward income shifting, we prove that the introduction of a tax allocation system based on FA in Europe could lead to a severe shift of economic substance to low-tax countries. Finally, we show that very high costs for deviating from the optimal pre-tax investment decision, as might be typical for traditional producing industries, result in low distortional effects for both tax systems. Accordingly, for very low costs for deviating from the optimal pre-tax resource-allocation the magnitude in distortions created by both systems is also equal, even though the level of distortion is maximally high under both systems.

As currently data on the status quo for income shifting via transfer prices under SA and on entrepreneurial investments after the introduction of FA are lacking, an analytical examination is important to understand and anticipate potential effects resulting from a switch in tax base allocation systems. The results of this paper are of particular interest for policy-makers. On an aggregated level, our results help anticipating the macroeconomic effects induced by introducing FA, especially against the background of the proposed CCCTB system and of Pillar One of the global tax reform. As we take a business perspective, our results may induce MNEs to reassess their recent locational investment decisions in the face of a potential future change in the applied tax base allocation system.

The next section consists of a brief review of the related literature. Section 3 presents the model with its assumptions and its set up. Moreover, we derive our results in Sect. 3 and graphically illustrate them. In Sect. 4 we discuss our results. In Sect. 5 we conclude.

## Literature

Two main streams of research are relevant to our research question. First, prior research examines the impact of taxation on investment decisions. In an early study, Johansson (1969) investigates the impact of income taxation on investment decision with a focus on neutrality of a corporate tax system. Similar to our approach, he measures distortions by comparing pre-tax with after-tax profitability. More precisely, he creates a rank order of projects after taxation and compares them with the pre-tax rank order. He finds that in practice conditions for neutral tax systems are almost never fulfilled. However, he is not concerned with international locational decisions and different tax regimes.

Our research idea shows some parallels with the long-lasting tax competition literature in public finance that is based on a SA setting. The analysis of neutrality of a tax system with respect to investment decisions from a business perspective is the other side of the coin of countries competing for tax bases from a public finance angle. The closest-related study to our work is by Haufler and Wootton (1999). In their model, based on a SA system, they assume that an MNE sells its output on a market with imperfect competition. Since there are transport costs, the firm will prefer to locate in the larger market. This gives rise to a locational rent for the large country, which may be exploited by tax policy, meaning that the large country may raise higher taxes than the small country. Nonetheless, the small country will still attract investments. The basic mechanism determining their outcomes are also decisive in our analysis: different market sizes mirrored by different demand intensities accompanied by different tax rates and costs for selling products to foreign markets. However, their goal is to investigate the optimal taxation strategy by the countries. Even though investment decisions and their distortions are indirectly important for their outcomes, they do not explicitly investigate them. An important element to our model that is missing in Haufler and Wootton (1999), but is considered by Haufler and Schjelderup (2000), is the possibility to shift income via transfer prices. In one variation in their model for optimal taxation, Haufler and Schjelderup (2000) allow firms to conduct foreign direct investment and manipulate transfer prices in order to shift income to low tax jurisdictions. In this case, countries reduce their profit tax rates in order to limit profit shifting to low tax countries. The opportunity for profit shifting thus acts as a restriction on profit taxation. In the presence of such a restriction, it is optimal to distort the investment decision. Their results speak to ours in so far as transfer pricing plays an important role for investment decisions and causes distortions. However, in contrast to our study, they model transfer prices as an arbitrarily manipulatable decision variable. In an empirical investigation, Overesch (2009) examines a related research question. He analyses whether MNEs’ real investment in high-tax countries is affected by income-shifting opportunities. Based on a panel of German inbound investments, he finds evidence that investments in high-tax countries increase if the MNE is able to shift income to low-tax jurisdictions. Furthermore, Grubert (2003) finds empirical evidence that companies with good income shifting opportunities prefer to invest in countries with either very high or very low statutory tax rates. The results of both studies are consistent with our findings that under SA income shifting opportunities affect locational decisions for the factor allocation.

Only a few studies focus on investment incentives in an FA setting. Gordon and Wilson (1986) analytically investigate how FA affects companies’ investment incentives. They conclude that a three-factor apportionment formula de facto creates three different taxes. Furthermore, largely in line with our results, they find that a formula consisting of assets, labor and sales creates incentives to produce in low-tax countries and sell in high-tax countries. Goolsbee and Maydew (2000) find in a study based on US data, that, on average, a reduction in the formula factor weight of payroll from one-third to one-quarter increases manufacturing employment by around 1.1%. In a similar vein, Riedel (2010) and Eichfelder et al. (2018) analyze how companies react to FA focusing on the German local business tax with payroll expense as the exclusive apportionment factor. Riedel (2010) finds that MNEs distort their payroll costs in favor of low-tax locations. Eichfelder et al. (2018) find evidence for significant tax effects on labor input at the intensive margin. In addition, they find evidence of an indirect FA spillover effect on capital investment. Our findings lend further support to these results in that MNEs adjust their business structure to the tax environment.

The second main stream of research relevant to our study focuses on the comparison of SA and FA with respect to various aspects. Many studies chose a public finance perspective. In line with our approach, Nielsen et al. (2010) model both tax systems and compare them with respect to basic properties, such as their impact on capital formation, input choices and transfer pricing. They focus on fiscal externalities and welfare effects created under both systems. Nielsen et al. (2003) investigate the effects of a switch from SA to FA on income shifting via transfer pricing in a setting with imperfect competition. Assuming that transfer pricing is still existent under FA in order to determine the sales factor, they conclude that a switch from SA to FA may even strengthen profit shifting activities via transfer pricing by MNEs. However, since in the recent CCCTB proposal by the European Commission transfer pricing is completely eliminated, we consistently consider transfer pricing in our model set-up only for SA. Gresik (2016) investigates the consequences on firm behavior and tax revenues in case firms can choose between FA and SA. Like we do, he explicitly models the possibility of shipping intermediate products from one country to the other; but in contrast to us, he does not assume costs for the shipment. However, he assumes costs for auditing the transfer pricing strategy in case of intergroup trade under SA, which could be re-interpreted—in a wide sense—as shifting costs. But, there are no comparable costs under FA in his model. Thus, his setting is not appropriate for deriving conclusions about the distortive power of each system. Taking a business perspective, Martini et al. (2012) investigate the impact of both tax base allocation regimes on production and investment decisions of MNEs. They focus on the internal decision-making process of MNEs by involving strategic considerations of subsidiary managers. They distinguish between centralized and decentralized decision structures within the MNE. In contrast to our results, they find that in a centralized decision structure, taxation is irrelevant for investment decisions under SA. This deviation in results is rooted in the definition of the (pre-tax) benchmark. Their benchmark is not based on optimal pre-tax investment decisions (i.e. sale opportunities) but instead on providing optimal incentives for managers, meaning that in their benchmark case, transfer prices are already included. Under FA they find, that the MNE’s internal organization has a decisive impact on the effects of the tax system on investment and production decisions. On a more abstract level, our results confirm that FA has some impact on the MNE’s investment decisions. Whereas all of the four mentioned studies incorporate model elements important to our study, none of them focuses on the distortive power of tax base allocation systems.

To conclude, comprehensive research has been conducted on the impact of taxation on locational investment decisions under SA and to a lesser extent also under FA. However, no study explicitly measures the level of distortion induced by each tax base allocation system and thus they have not been compared. Although there is some literature comparing the specific properties of SA and FA, the studies disregard the distortive power they have on locational investment decisions. Our study aims to fill this gap.

## Model

### Assumptions

An entrepreneur has established a profitable business, consisting of two entities in country A and country B. We assume a fixed investment fund which can be invested in assets and workforce.^2^ The target markets for the services/products are country A and country B. The investment is assumed to be continuous with respect to the allocation of funds. Thus, the entrepreneur can split the investment budget in every way and invest a portion in asset and in workforce in country A and the rest in country B. We assume that the two resulting entities are fully affiliated and build a multinational entity (MNE).

The investment budget the MNE expends for workforce is $hv$ and for assets is $inv$. The input factors $hr$ and $inv$ $\frac{hr}{inv}$ between the input factors must not (but can) be 1.^3^

Sales are a valued quantity as well. For simplicity we set the sales $$S = 1$$.We focus solely on profitable investments, thus $$hr + inv < 1$$ holds.^4^ Summarizing the overall costs for assets and workforce by $$c_{G}$$$$(c_{G} = inv + hr)$$, the implicit pre-tax return $$r$$ of the MNE is

1 $$r = \frac{1}{{c_{G} }} - 1.$$

As a matter of definition, note for later use, that $c_{G}$ does not include non-optimality costs.

The specific demand for the final products in countries A and B is exogenously given. The level of investment in the input factors $hr$ and $inv$ per country is determined by a specific pre-tax optimum. In order to single out pure tax effects, we abstract from differences in productivity in country A and B.

The variable $a$ or $(1 - a)$, respectively describes the relative demand for the final products in country A or B $a \in \left( {0,1} \right)$. In the pre-tax case it is optimal for the MNE to produce there where the customers are in our simplified setting. This assumption implies equal marginal contribution of the partial investments. Thus, the pre-tax optimum for the investment in assets and workforce is consequently given by $a$ as well. With respect to the demand, the two countries may deviate from each other.^5^ The distribution policy of the MNE is assumed to stipulate that it sells its products to local customers in country A and B only and exclusively via the local entity. This distribution structure based on exclusive territory distribution agreements is a common approach in several industries, e.g. in the petrol or automotive industry.

The core of our analysis is to determine a potential deviation from the optimal pre-tax factor allocation induced by the respective tax system. Considering taxation, the entrepreneur invests the share $\alpha$ in total assets in country A $\left(\hspace{0.33em}inv\right)$ and the share $\left(1-\right)$ in country B$\left(\left(1-\right)\hspace{0.33em}inv\right)$. Similarly, she invests the share $\rho$ in overall workforce in country A $\left( {\rho \;hr} \right)$ and the share $(1 - \rho )$ in country B $\left( {\left( {1 - \rho } \right)\;hr} \right)$, respectively. Consequently, the after-tax optimum $\alpha$ may differ from the pre-tax optimum $a$. Then, resource-allocation compared to the pre-tax-setting is non-optimal. This will cause costs compared to the pre-tax optimum. In the following we summarize these costs under the term “non-optimality costs” $C_{non - opt}$.^6^

In the context of our analysis, we do not consider a general equilibrium-model feasible. Rather, we consider a quadratic non-optimality cost function to be appropriate. The main feature of this cost function in the context of our analysis is, that any deviation of the pre-tax optimum causes non-optimality costs.

From an economic perspective these non-optimality costs may have diverse origins. If the MNE e.g. sells cars in country A and B but offers warranty services by tax reasons only in country A, this is inconvenient for customers in country B and probably costly for the entity, as cars must be transferred for warranty operations from B to A and retour. Moreover, costs for keeping customers mobile may arise. These costs are avoidable, if an optimal structure of the MNE is established. Any suboptimal structure of the MNE may e.g. cause additional governance costs, consulting expenses, translation costs, etc. We assume that these non-optimality costs are overhead costs that cannot directly be attributed to creating a product or service. Nevertheless, tax advantages may outweigh these costs and may justify them compared to a pre-tax setting. Thus, comparing tax-advantages of SA and FA with these non-optimality costs is the core of our model.

We justify quadradic non-optimality costs in a more formal way by referring to a Cobb–Douglas production function with input-factors $f_{1}$ and $f_{2}$:

2 $$Y = f_{1}^{\varepsilon } f_{2}^{1 - \varepsilon }$$

*Y* represents the output quantity, the input factors $f_{1}$ and $f_{2}$ may represent input factors like domestic and foreign production capacities. The factor $\varepsilon$ determines the factor-elasticity.

Assuming linear factor costs $c_{1}$ and $c_{2}$, overall production costs are given by

3 $$C_{pre} = c_{1} f_{1} + c_{2} f_{2} .$$

As the output is constant, a cost minimizing approach maximizes expected profit. Hence, in optimum the relation of marginal factor costs must be equal to the factor’s rate of substitution. Assuming $\varepsilon = 0.5$—what is not crucial for our analysis—leads to optimal input factor quantities of

4 $$f_{1}^{*} = \frac{1}{{\sqrt {{{c_{1} } \mathord{\left/ {\vphantom {{c_{1} } {c_{2} Y^{2} }}} \right. \kern-0pt} {c_{2} Y^{2} }}} }}\;{\text{and}}\;f_{2}^{*} = Y^{2} \sqrt {{{c_{1} } \mathord{\left/ {\vphantom {{c_{1} } {c_{2} Y^{2} }}} \right. \kern-0pt} {c_{2} Y^{2} }}} .$$

Overall production costs in pre-tax optimum are

5 $$C_{pre}^{*} = c_{1} f_{1}^{*} + c_{2} f_{2}^{*} .$$

For our analysis it is essential, which costs occur, if the entity deviates from the optimal factor allocation in (4). In order to show these effects of non-optimality we introduce $\delta$ as a deviation of $f_{1}^{*}$. Then, $f_{1} = f_{1}^{*} + \delta$ holds instead of $f_{1} = f_{1}^{*}$. The non-optimality costs $C_{non - opt}$ then are

6 $$C_{\delta } - C_{pre}^{*} = C_{non - opt} = \frac{{c_{1} \delta^{2} \sqrt {{{c_{1} } \mathord{\left/ {\vphantom {{c_{1} } {c_{2} Y^{2} }}} \right. \kern-0pt} {c_{2} Y^{2} }}} }}{{1 + \delta \sqrt {{{c_{1} } \mathord{\left/ {\vphantom {{c_{1} } {c_{2} Y^{2} }}} \right. \kern-0pt} {c_{2} Y^{2} }}} }}.$$

As the extend of deviation $\delta$ appears quadratic in the numerator but with power one in the denominator, non-optimality costs are not symmetric around optimum. But asymmetry declines with increasing *Y*. Hence, using a quadratic non-optimality cost function for deviations of a pre-tax optimum from an economic perspective is a reasonable proxy for the course of Cobb–Douglas non-optimality costs, if *Y* is reasonable high in relation to ${{c_{1} } \mathord{\left/ {\vphantom {{c_{1} } {c_{2} }}} \right. \kern-0pt} {c_{2} }}$. For other scenarios the quadratic non-optimality cost function—e.g. if optimal factor allocation is dominated by one factor due to superior elasticity or low costs—is not a sufficiently good proxy. Hence, we emphasize that our analysis does not provide quantitative insights into these specific scenarios.

**Fig. 1:**
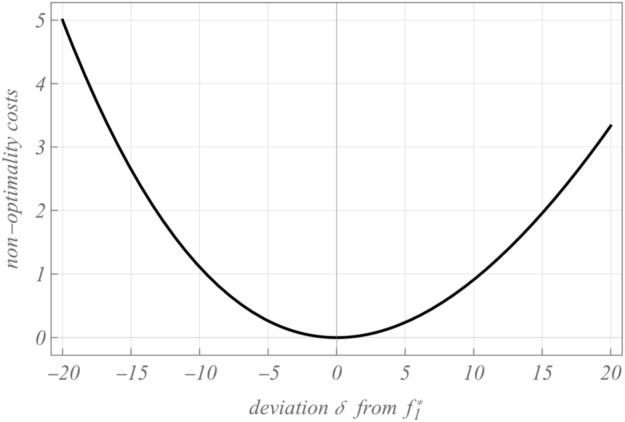
Non-optimality costs

Figure 1 illustrates, that a quadratic non-optimality function is a reasonable proxy for non-excessive deviations $\delta$ for parameter setting $\left\{ {Y \to 100,c_{1} \to 1,c_{2} \to 1} \right\}$.

Overall, we consider a quadratic cost function for non-optimality costs to be reasonable, although we are aware of the limitations. Thus, we incorporate non-optimality effects into our model in terms of a quadratic cost function. Accordingly, in our model non-optimality costs disproportionally increase with increasing deviations from the optimum regardless the direction of deviation:

7 $$C_{non - opt} = c_{d} \left( {\left( {a - \alpha } \right)^{2} + \left( {a - \rho } \right)^{2} } \right).$$

$c_{d}$ represents the cost parameter for deviating from the pre-tax optimum $(c_{d} > 0)$. For reasons of simplicity and in order to keep our model traceable, we assume that the costs for deviating from the pre-tax optimum are similar for the investment in assets and workforce.

For clarification, consider the following example: Let us assume the optimal pre-tax investment decision would be $a$ = 0.5, meaning that sales, asset and workforce are distributed equally between countries. Sales are normalized to one $\left(S=1\right)$. By assumption, the overall investment budget is ${c}_{G}=0.8$. Assuming the MNE is operating in a human-capital intense industry, the investment budget is allocated between asset and workforce as follows: $hr=0.6,inv=0.2$. Thus, in the pre-tax case total investment in assets in country A is $a*inv=0.5*0.2=0.1$ and in workforce is $a*hr=0.5*0.6=0.3$. As $a$=0.5, the same investment strategy holds for country B.

Taxation may distort the pre-tax investment decision. The optimal investment in assets and workforce per country will no longer be equal to $a$ but is after-tax given by $\alpha$ and $\rho$. Let us assume the optimization reveals that $\alpha =0.4$ and $\rho =0.2$. Thus, after-tax, in country A total investment in assets is $\alpha *inv=0.4*0.2=0.08$ and in workforce is $\rho *hr=0.2*0.6=0.12$. For country B holds for investment in assets $\left(1-\alpha \right)*inv=0.6*0.2=0.12$ and for investment in workforce $\left(1-\rho \right)*hr=0.8*0.6=0.48$. Thus, due to taxation, real activity is shifted in this example from country A to country B. This change in the structure of the MNE for tax purposes is causing non-optimality costs (see Eq. (7)), amounting to ${C}_{non-opt}=0.1$ assuming that ${c}_{d}=1$. In the most extreme case, the model theoretically allows for constellations in which all assets are located in one country and the whole workforce in the other (e.g. $\alpha =1$ and $\rho =0$). If such an allocation of input factors would be optimal, then the introduction of taxation would have caused a pure two-stage production process, in which e.g. all physical products are produced in one country and e.g. the labor-intense marketing is offered in the other.

Furthermore, the analysis is based on the following tax assumptions: Both countries levy corporate taxes on the entities’ profits. The tax rates are assumed to be identical under FA and SA and range between 0 and 100% $\tau_{A} \wedge \tau_{B} \in \left( {0,1} \right)$.^7^ We define the tax rate differential $\Delta \tau = \tau_{B} - \tau_{A}$. In case of $\tau_{A} > \tau_{B}$, A is the high-tax country. We abbreviate $\tau_{A} > \tau_{B}$ with $\Delta \tau^{ - }$ and use the notation $\Delta \tau^{ + }$, if B is the high-tax country.

If the funds are invested in assets (e.g. machinery), they are immediately and fully depreciable as we apply a one-period setting.

In the following sections we determine the optimal after-tax allocation of investment funds in order to determine the level of distortion caused by each tax system. It is important to emphasize, that distortional effects result from comparing tax advantages on the one hand and non-optimality costs on the other hand.

### Optimal factor allocation under separate accounting

With its overall production, the MNE perfectly meets the demand of customers aggregated over both countries, meaning that as many products are produced by the MNE as demanded by final customers over both countries.

As each entity sells products only to local customers, the relative demand per country is equal to the relative volume of sales to customers of each entity in that country. Thus, the relative volume of sales to customers is represented by $a$ as well. Note, however, that the relative after-tax production volume per entity determined by $\alpha$ for the input factor assets and $\rho$ for the input factor workforce is not necessarily equal to its relative sales volume to customers $a$. Due to tax planning considerations, the volumes of products produced and products sold may differ in each country. If such a deviation occurs, the overproducing entity internally sells products to the underproducing entity. Note that in sum the two entities meet exactly the sum of demand in the two countries. Our model does not allow for stock keeping.

The following Table 1 summarizes and illustrates the cases in which the entity in country A or B is a supplier/demander.

**Table 1:** Relationship between sold and produced products per country

	Country A (Consequences for country B follow accordingly)	
Production capacities for assets and workforce	Assets $\alpha$	Workforce $\rho$
Share of demand = share of products sold	a	
Direction of intercompany trade (note that $\alpha$ and $\rho$ do not depend on each other)	$\alpha > a$ Overproduction in country A, underproduction in country B, transfer of products from A to B	$\rho > a$ Excessive workforce in country A, lacking workforce in country B, transfer of services/IP from A to B
	$\alpha < a$ Overproduction in country A, underproduction in country B, transfer of products from A to B	$\rho > a$ Lacking workforce in country A, excessive workforce in country B, transfer of services/IP from B to A

If products/services are traded cross-border between MNEs’ entities, transfer prices need to be applied under SA. Transfer prices might range between production costs and market prices in our setting. Thus, the lower limit for transfer pricing payments is determined by the intergroup trade valued at costs:

8 $$TP_{min} = \left( {{\text{hr }}\rho + {\text{inv }}\alpha } \right) - \left( {{\text{hr }}a + {\text{inv }}a} \right).$$

If ${TP}_{min}$ is negative (positive), then entity A is net supplier (demander) of products/services.

The upper limit for valuing transfer pricing payments is determined by market prices that are equal to minimal transfer pricing payments valued at costs multiplied with the overall pre-tax return *r* of the group:

9 $$TP_{max} = \left( {1 + r} \right){ }TP_{min} .$$

The transfer pricing parameter $tp \in \left( {0,1} \right)$ determines the actual transfer pricing range. The overall transfer pricing payments $TP$ are then calculated as follows

10 $$\begin{aligned} TP & = TP_{{min}} + \left( {TP_{{max}} - TP_{{min}} } \right)tp \\ & = TP_{{min}} + (\frac{1}{{c_{G} }}TP_{{min}} - TP_{{min}} )tp \\ & = \frac{{\left( {tp\left( {c_{G} - 1} \right) - c_{G} } \right)\left( {a\;c_{G} - hr\rho - \alpha \;inv} \right)}}{{c_{G} }} \\ \end{aligned}$$

The parameter $tp$ determines which fraction of the overall profits are allocated to each entity. When $tp = 0$ no profits at all are shifted by transfer pricing, instead products/services are traded within the MNE on the basis of incurred costs. In the case when $tp = 1$, products/services are sold on the basis of market prices, meaning that the entire profits for the products sold internally are shifted to the entity selling the products. Note that the upper limit of the transfer pricing range is the market price without deducting the non-optimality costs. Reason is that in our setting the non-optimality costs need to be interpreted as negative synergy effects, that—in line with Z 1.157 of the OECD Transfer Pricing Guidelines—entities need to bear without receiving compensation for it. Negative synergy effects e.g. result when the complexity of business activities create bureaucratic hurdles that smaller and more flexible companies do not face. Our non-optimality costs capture exactly these costs of complexity that only occur as the MNE establishes an intertwined group structure by establishing intercompany trade. Note, that in the pre-tax setting, the entities are not intertwined at all and are fully independent from each other.

The allocation of assets and workforce in terms of $\alpha$ and $\rho$ influences the transfer price. Note, that for calculating $TP$ the shifted products/services and transfer pricing payments are already consolidated and are thus net with respect to the input factors assets $inv$ and workforce $hr$.

Equation (8) clarifies that in our model the transfer pricing payments are determined by (i) the transfer price itself and (ii) the occurrence of assets and workforce (hr ρ; inv α) per country. The more production capacities (asset and workforce) are located in a country, the more products are fabricated there. These products can be sold to the entity in the other country and trigger tax-optimized transfer pricing payments. In line with this modeling approach, empirical evidence indicates strongly that FDI is positively related to low tax rates (see (Mooij and Ederveen 2003) and (Davies 2021)). Thus, the extent of economic substance in a country determines the amount of received transfer pricing payments and thereby the share of profits taxed in a country. However, this mechanism is discussable for the linkage of the place of creating IP (occurrence of workforce) and the place of earning income from IP. Empirical evidence from the pre-BEPS area shows that MNEs located IP in tax havens where they basically had no economic substance (Griffith et al. 2014). However, in the aftermath of the OECD BEPS project, the increasing implementation of the nexus approach, of exit taxes and of deduction restrictions for royalty payments in national tax legislation causes MNEs to more and more give up on disentangling the location of creating IP (occurrence of workforce) and the location of holding IP and generating sales from them.

We assume that the MNE applies transaction-related standard transfer pricing methods.^8^ If the MNE is entirely free to choose the transfer price within this range, it will always end up setting the transfer price equal to the market price in our setting. However, the legal transfer pricing framework likely narrows this range. Especially after the national implementation of the OECD BEPS project, it is reasonable to assume that the transfer price cannot be set arbitrarily.^9^ As no costs are caused for transfer pricing,^10^ it is always optimal for the MNE to choose a transfer price at the end of the range accepted by the tax authorities. The tax rate differential determines which end of the range is optimal to choose.

Furthermore, non-optimality costs ${C}_{non-opt}$ are incurred for the cross-border trade between the entities. The split of the costs between countries/entities need to be determined under SA. It seems reasonable that the entities share the costs to some extent as both entities deviate from their optimal pre-tax investment structure in case non-optimality costs occur. We assume that the non-optimality costs are borne by the entities in relation to their optimal pre-tax investment decision $a$. That seems plausible as the bigger entity (before tax-driven distortions in the group structure) bears a relatively bigger share of the costs. A split of the non-optimality costs ${C}_{non-opt}$ according to the after-tax investment decisions $\alpha$ or $\rho$ would create incentives to further distort after-tax investment decisions which do not enter the entrepreneurial decision calculus in practice. The MNE has incentives to deduct as many non-optimality costs as possible in the high-tax country since no costs are linked to the allocation of non-optimality costs.

Under SA, the after-tax profits of entity A are calculated as follows:

11 $$\pi_{SA}^{A} = \left[ {a - hr\;\rho - inv\;\alpha + TP - a\;C} \right]\;\left( {1 - \tau_{A} } \right).$$

Accordingly, the after-tax profits of entity B are obtained by:

12 $$\pi_{SA}^{B} = \left[ {\left( {1 - a} \right) - hr\left( {1 - \rho } \right) - inv\left( {1 - \alpha } \right) - TP - \left( {1 - a} \right)C} \right]\left( {1 - \tau_{B} } \right).$$

Consequently, the overall after-tax profits of the MNE are given by:

13 $$\pi_{SA}^{{}} = \pi_{SA}^{A} + \pi_{SA}^{B} .$$

Differentiating the after-tax group profits $\pi_{SA}^{{}}$ with respect to the decision variables $\alpha$ and $\rho$ shows the effects of a marginal change in $\alpha$ and $\rho$ on the overall profits $\pi_{SA}^{{}}$:

14 $$\partial \pi_{SA}^{{}} \left( \alpha \right) = 2\left( {a - \alpha } \right)c_{d} \left( {1 - \tau_{D,SA} } \right) + \frac{{\Delta \tau \;\pi_{G} \;inv\;tp}}{{c_{G} }},$$

15 $$\partial \pi_{SA}^{{}} \left( \rho \right) = 2\left( {a - \rho } \right)c_{d} \left( {1 - \tau_{D,SA} } \right) + \frac{{\Delta \tau \;\pi_{G} hr\;tp}}{{c_{G} }},$$

with.

$\tau_{D,SA}$ being the average tax rate weighted with sales before profit-shifting:

16 $$\tau_{D,SA} = a\tau_{A} + \left( {1 - a} \right)\tau_{B} = \Delta \tau - a\Delta \tau + \tau_{A} .$$

and with.

$\pi_{G} = 1 - hr - inv$ being the total pre-tax profit of the MNE (before non-optimality costs).

A change in $\alpha$ and $\rho$ affects the marginal after-tax non-optimality costs (First summand of Eqs. (14) and (15)) and the marginal after-tax transfer pricing payments (second summand of Eqs. (14) and (15)). Talking about the first summand of Eqs. (14) and (15), the expression in the second brackets in eqs. (14) and (15) (i.e. $1 - \tau_{D,SA}$) capturing the net tax factor for a marginal change in non-optimality costs is always positive $.$ Incorporating the definition of Eq. (16) for ${\tau }_{D,SA}$ clarifies the effects resulting from the net tax factor. In more detail, this expression captures for a changing $\alpha$ or $\uprho$ i) the marginal after-tax non-optimality costs in country A $(1 - \tau_{A} )$, ii) the marginal net tax effect from a changed allocation of investment expenses in both countries $(\Delta \tau )$, and iii) the marginal portion of complexity costs deductible in country A $(a\Delta \tau )$. Talking about the second summand of Eqs. (14) and (15), it is obvious that a change in $\alpha$ or $\rho$ has no impact on transfer pricing payments if the tax rates are similar in both countries $(\Delta \tau = 0)$. With no tax rate differential, there is no incentive for the MNE to shift profits via transfer pricing.

In the optimum $\upalpha _{SA}^{*}$ and/or $\rho_{SA}^{*}$, the marginal utility from a change in $\alpha$ and/or $\uprho$ equals the marginal costs (first summand equals second summand in Eqs. (14) and/or (15)). Which summand represents the marginal costs or the marginal utility, respectively, depends on the sign of the tax rate differential.

**Lemma 1:** Under separate accounting the optimal after-tax investment decisions $\alpha_{SA}^{*}$ and $\rho_{SA}^{*}$ are given by

17 $$\alpha_{SA}^{*} = a + \frac{{\Delta \tau \;\pi_{G} \;inv\;tp}}{{2c_{d} c_{G} \left( {1 - \tau_{D,SA} } \right)}},\rho_{SA}^{*} = a + \frac{{\Delta \tau \;\pi_{G} \;hr\;tp}}{{2c_{d} c_{G} \left( {1 - \tau_{D,SA} } \right)}},$$

*as long as holds:* $\alpha_{SA}^{*} \in \left[ {0,1} \right]$ *and* $\rho_{SA}^{*} \in \left[ {0,1} \right]$ *.*

The distortional effects created by separate accounting are given by $\delta_{SA,\alpha }$ and $\delta_{{SA,\uprho }}$ as the difference between the optimal pre-tax investment decision $a$ and the optimal after-tax investment decision for each input factor $\alpha_{SA}^{*}$ and $\rho_{SA}^{*}$ $\left( {\delta_{SA,\alpha } = a - \alpha_{SA}^{*} ;\delta_{SA,\rho } = a - \rho_{SA}^{*} } \right)$, i.e.:

18 $$\delta_{SA,\alpha } = - \frac{{\Delta \tau \;\pi_{G} inv\;tp}}{{2c_{d} c_{G} \left( {1 - \tau_{D,SA} } \right)}},\delta_{{SA,\uprho }} = - \frac{{\Delta \tau \;\pi_{G} hr\;tp}}{{2c_{d} c_{G} \left( {1 - \tau_{D,SA} } \right)}}.$$

The optimal factor allocation $\alpha_{SA}^{*}$ and $\rho_{SA}^{*}$ for the input factors assets $inv$ and workforce $hr$ are shown in Eq. (17) as the deviation from the optimal pre-tax investment decision $a$. The fractions, i.e. the deviation from the optimal pre-tax investment decision $a$ in Eq. (17), is positive or negative, depending on the sign of the tax rate differential (sign of $\Delta \tau$). The denominator is always positive.

Border solutions may occur, i.e. a change in parameters does not result in an optimal investment decision that is bigger than one or smaller than zero ($\alpha_{SA}^{*} \in \left[ {0,1} \right]$ and $\rho_{SA}^{*} \in \left[ {0,1} \right]$). Inner solutions for $\alpha_{SA}^{*}$ (and $\rho_{SA}^{*}$) occur in a case where the tax rate differential $\Delta \tau$ lies within the following limits:

19 $$\left\{ {\Delta \tau_{SA}^{0} = - \frac{{2a\left( {1 - \tau_{A} } \right)c_{d} c_{G} }}{{\pi_{G} \;inv\;tp - 2a\left( {1 - a} \right)c_{d} c_{G} }},\Delta \tau_{SA}^{1} = \frac{{2\left( {1 - a} \right)\left( {1 - \tau_{A} } \right)c_{d} c_{G} }}{{2\left( {1 - a} \right)\left( {1 - a} \right)c_{d} c_{G} + \pi_{G} \;inv\;tp}}} \right\}.$$

As seen by Eq. (18), the deviation from the optimal pre-tax investment decision $a$ is always negative for $\Delta \tau^{ - }$ ($\upalpha _{SA}^{*} < a,\uprho _{SA}^{*} < a$, thus $\delta_{SA,\alpha } > 0$ and $\delta_{{SA,\uprho }} > 0$). Note, that the distortions $\delta_{SA,\alpha }$ and $\delta_{{SA,\uprho }}$ itself are independent of the optimal pre-tax investment decision $a$. Thus, an increase in $a$ raises the optimal after-tax investment decisions $\alpha_{SA}^{*}$ and $\rho_{SA}^{*}$ simply by the amount of the increase.

For $\Delta \tau^{ - }$, the after-tax investment decisions $\alpha_{SA}^{*}$ and $\rho_{SA}^{*}$ decrease with higher transfer prices $tp$ (see (17)). Higher transfer prices $tp$ indicate a shifting of higher portions in profits via cross-border trade. Thus, higher transfer prices create incentives to produce more in the low-tax country B since trading the excessive products cross-border leads to higher expenses in the high-tax country A and to higher profits in the low-tax country B. Consequently, with increasing $tp$, the distortions become more severe (see (18)). Transfer pricing possibilities and the distortion of the factor allocation with respect to their location have a mutually reinforcing effect.

Higher levels of assets (*inv*) and/or workforce (*hr*) results in decreasing profits. As deviations costs are not driven by profits, they remain constant. Due to decreasing tax savings with decreasing profits, the incentive to deviate from the pre-tax optimum is decreasing.

An increasing deviation cost parameter $c_{d}$ results in a stronger incentive to shrink the difference between the optimal pre-tax investment decision $a$ and the optimal after-tax investment decisions $\alpha_{SA}^{*}$ and $\rho_{SA}^{*}$. As for $\Delta \tau^{ - }$, the optimal after-tax investment decisions $\alpha_{SA}^{*}$ and $\rho_{SA}^{*}$ are always lower than the optimal pre-tax investment decision $a$ ($\alpha_{SA}^{*} < a$ and $\uprho _{SA}^{*} < a$), an increase in the non-optimality costs parameter $c_{d}$ leads to higher optimal after-tax investment decisions $\alpha_{SA}^{*}$ and $\rho_{SA}^{*}$ and to smaller distortions.

The effect of a bigger tax rate differential in case of $\Delta \tau^{ - }$ on the optimal after-tax investment decisions $\alpha_{SA}^{*}$ and $\rho_{SA}^{*}$ is obvious: An increase in the tax rate differential $\Delta \tau^{ - }$ leads to stronger incentives to set up a tax-optimal structure, meaning that the MNE invests more in the low-tax country B $.$ Consequently, the distortions increase with increasing tax rate differential $\Delta \tau^{ - }$.

### Optimal factor allocation under formula apportionment

For apportioning the consolidated tax base of an MNE under FA, the design of the formula is decisive. The well-known Massachusetts Formula consists of the equally weighted factors of assets, labor and sales (notation here in line with our model set-up, subscript *i* represents entity $i \in \left\{ {A,B} \right\}$):

20 $$f_{i} = \frac{1}{3}\left( {\frac{inv_i}{{inv}} + \frac{hr_i}{{hr}} + \frac{S_i}{S}} \right).$$

The Massachusetts Formula is—with a small deviation—the proposed formula for apportioning the tax base of European MNEs under the CCCTB system.^11^ The Massachusetts Formula was originally used by almost all states in the US to apportion the consolidated tax base of national groups to the entities. However, whereas under the proposed CCCTB system the factors are weighted equally, in the US there is room for deviation from these weights. States tend to give more weight to the sales factor and distribute the remaining weights equally across the asset and the labor factor. We base our analysis on equally weighted factor weights in line with the Massachusetts Formula.

Applied to our model set-up, the share of the group tax base that is allocated to country A $(f_A)$ is determined as

21 $$f_A = \frac{1}{3}\left( {\frac{a\;S}{S} + \frac{\alpha \;inv}{{inv}} + \frac{\rho \;hr}{{hr}}} \right) = \frac{1}{3}(a + \alpha + \rho )$$

Consequently, the share allocated to country B is $f_B = 1 - f_A$, respectively. Note that absolute values for sales $S$, assets $inv$ and workforce $hr$ have no impact on the formula. The overall after-tax group-profits under FA $\pi_{FA}^{{}}$ are given by

$$\pi_{FA} = \left[ {1 - \tau_{B} + \frac{1}{3}\Delta \tau \left( {a + \alpha + \rho } \right)} \right]\left[ {\pi_{G} - C_{non - opt} } \right]$$

22 $$= \frac{1}{3}\left[ {3\left( {1 - \tau_{A} - \Delta \tau } \right) + \Delta \tau \left( {a + \alpha + \rho } \right)} \right]\left[ {\pi_{G} - c_{d} \left( {(a - \alpha )^{2} + (a - \rho )^{2} } \right)} \right]$$

The term in the first squared brackets in the first row of Eq. (22) represents the impact of taxation, the term in the second squared brackets stands for the MNE’s pre-tax profits after deducting complexity costs. The tax term in the first squared brackets in the first row of Eq. (22) shows the after-tax profits if the MNE would solely be taxed in country B (i.e. $1-{\tau }_{B}$) plus the deviation from that tax base allocation as some fraction of the tax base is allocated to and taxed in country A (i.e.$\Delta \tau \frac{1}{3}\left(a+\alpha +\rho \right)$).

Note that the non-optimality costs ${C}_{non-opt}$ affect the pre-tax profits but have no impact on the allocation of the tax base. Moreover, as the non-optimality costs are simply expenses that do not affect either of the apportionment factors, the allocation of these costs between the entities does not play a role under FA. The concept of transfer pricing is not applied under FA.^12^

To determine FOC for optimal factor allocation we derive after-tax group profits $\pi_{FA}^{{}}$ with respect to the action variables $\alpha$ and $\rho$:

23 $$\begin{aligned} \frac{{\partial \pi_{FA}^{{}} }}{\partial \alpha } & = \frac{{\partial \pi_{FA}^{{}} }}{\partial \rho } \\ = \frac{{\Delta \tau \pi_{G} }}{3} - \frac{1}{3}\left( {\Delta \tau \left( {2\alpha \rho + 3\left( {\alpha - 2} \right)\alpha + \rho^{2} } \right) - c_{d} \left( {2a\left( {\Delta \tau \left( {\alpha + 2\rho - 3} \right) - 3\tau_{A} + 3} \right) - 6\alpha \left( {1 - \tau_{A} } \right)} \right)} \right) \\ \end{aligned}$$

The effects of a change in allocation in assets $\alpha$ and workforce $\rho$ on the after-tax profits are more complex under FA than under SA.

The action variables $\alpha$ and $\rho$ affect directly the relative allocation of the tax base with respect to the apportionment factors assets and labor. An increase in $\alpha$ and/or $\rho$ means that relatively more of the MNE’s tax base is taxed in country A. Consequently, under FA, a change in $\alpha$ and/or $\uprho$ does not only directly affect the pre-tax non-optimality costs but also directly affects the net tax payments on the non-optimality costs and on the remaining profits by the changed allocation of the tax base. The net tax factor for a marginal change in non-optimality costs under FA is captured by $\tau_{D,FA} = \frac{1}{3}\left[ {\Delta \tau \left( {a + \alpha + \rho } \right) - 3\left( {\tau_{A} + \Delta \tau - 1} \right)} \right]$. In contrast, under SA the net tax factor on the non-optimality costs $\tau_{D,SA}$ is not influenced by the decision variables $\alpha$ and $\rho$ at all. The technical reason is, that due to the product rule, cross-effects of non-optimality costs and the group’s tax rate occur.

As action variables $\alpha$ or $\rho$ impact only the apportionment factor “assets” or “labor”, which is weighted by one-third each, the change in the after-tax profits (before non-optimality costs) is divided by 3 (first fraction in Eq. (23)).

**Lemma 2:** Under formula apportionment, the optimal investment decisions are *given by*^13^

24 $$\alpha_{FA}^{*} = \rho_{FA}^{*} = \frac{{\sqrt {c_{d} \left( {3c_{d} \left( { - a\Delta \tau + \tau_{A} + \Delta \tau - 1} \right)^{2} + 2\Delta \tau^{2} \pi_{G} } \right)} }}{{2\sqrt 3 \Delta \tau c_{d} }} + \frac{{a\Delta \tau + \tau_{A} + \Delta \tau - 1}}{2\Delta \tau },$$

as long as holds: $\alpha_{FA}^{*} \in \left[ {0,1} \right]$* and *$\rho_{FA}^{*} \in \left[ {0,1} \right]$*.*

The distortions $\delta_{FA}$ given by the difference between the optimal pre-tax investment decision $a$ and the optimal after-tax investment decision for the input factors ${\alpha }_{FA}^{*}={\rho }_{FA}^{*}$, are calculated as follows:

25 $$\delta_{FA} = a + \frac{{1 - \left( {a + 1} \right)\left( {\tau_{A} + \Delta \tau } \right) + a\tau_{A} }}{2\Delta \tau } - \frac{{\sqrt {c_{d} \left( {3c_{d} \left( {\left( {1 - a} \right)\Delta \tau + \tau_{A} - 1} \right)^{2} + 2\Delta \tau^{2} \pi_{G} } \right)} }}{{2\sqrt 3 \Delta \tau c_{d} }}.$$

In contrast to the optimal factor allocation under SA ($\alpha_{SA}^{*}$ and $\rho_{SA}^{*}$), those under FA are always similar ($\upalpha _{FA}^{*} =\uprho _{FA}^{*}$), see Eq. (24). Like under SA, the optimal after-tax investment decisions $\alpha_{FA}^{*}$ and $\rho_{FA}^{*}$ are always lower than the optimal pre-tax investment decision $a$ ($\alpha_{FA}^{*} < a$ and $\rho_{FA}^{*} < a$) for $\Delta \tau^{ - }$. Again, the previously described effects only occur as long as inner solutions for $\alpha_{FA}^{*}$ and $\rho_{FA}^{*}$ are found. That is the case if the tax rate differential $\Delta {\tau }_{FA}$ lies within the following limits:

26 $$\left\{ {\Delta \tau_{FA}^{0} = - \frac{{6a\left( {1 - \tau_{A} } \right)c_{d} }}{{\pi_{G} - 6ac_{d} }},\Delta \tau_{FA}^{1} = \frac{{6\left( {1 - a} \right)\left( {1 - \tau_{A} } \right)c_{d} }}{{\pi_{G} }}} \right\}.$$

Following the same mechanism as described under SA, an increase in the deviation cost factor $c_{d}$ tightens the optimal pre-tax investment decision up with the optimal after-tax investment decision (see Eq. (24)). Consequently, for $\Delta \tau^{ - }$, an increase in $c_{d}$ leads to an increase in $\alpha_{FA}^{*}$ and $\rho_{FA}^{*}$ ($\partial \delta_{FA} \left( {c_{d} } \right) < 0$ and $\partial \alpha_{FA}^{*} \left( {c_{d} } \right) > 0$ and $\partial \rho_{FA}^{*} \left( {c_{d} } \right) > 0$).

A change of the optimal pre-tax investment decision $a$ has more complex effects on the optimal after-tax investment decisions as it has under SA. Whereas under SA the change only affects the level of the optimal investment decisions $\alpha_{SA}^{*}$ and $\rho_{SA}^{*}$ (see Eq. (17)) but has no influence on taxation, it affects taxation under FA as well (see Eq. (25)). An increase in $a$ leads to a change in pre-tax non-optimality costs and in changed tax consequences for these costs (see Eq. (22)). In the base case, a higher optimal pre-tax investment decision $a$ translates into higher marginal tax payments as a higher portion of the apportionment factor sales is allocated to the high-tax country. Moreover, from a pre-tax perspective, an increase in $a$ leads to higher non-optimality costs (remember in the base case holds: $\alpha_{FA}^{*} < a$ and $\rho_{FA}^{*} < a$). The entrepreneur reacts to an increase in $a$ by increasing after-tax investment into country A (increase $\alpha_{FA}^{*}$ and $\rho_{FA}^{*}$) in order to adjust (i.e. reduce) the non-optimality costs. To reach the optimum she balances the reduction in non-optimality costs with the increased tax payments resulting from an increase in $\alpha_{FA}^{*}$ and $\rho_{FA}^{*}$. As the tax rate is below 100%, the distortions are increased by an increase in $a$ as well ($\partial \alpha_{FA}^{*} \left( a \right) > 0$ and $\partial \rho_{FA}^{*} \left( a \right) > 0$ and $\partial \delta_{FA} \left( a \right) > 0$. The effect of changes in the tax rate differential in case of $\Delta \tau^{ - }$ on the optimal after-tax investment decisions $\alpha_{FA}^{*}$ and $\rho_{FA}^{*}$ follows the same line of argumentation than for their change under SA: An increase in the tax rate differential $\Delta \tau^{ - }$ leads to stronger incentives to set up a tax-optimal structure, meaning that the MNE invests more in the low-tax country B ($\partial \alpha_{FA}^{*} \left( {\Delta \tau } \right) < 0$ and $\partial \rho_{FA}^{*} \left( {\Delta \tau } \right) < 0$). Consequently, the distortions increase with increasing tax rate differential $\Delta \tau$ ($\partial \delta_{FA} \left( {\Delta \tau } \right) > 0$) for $\Delta \tau^{ - }$.

### Comparison of distortional effects under SA and FA

The goal of this study is to investigate which tax base allocation system leads to stronger distortions under certain conditions. Thus, the difference $\Delta \delta$ between the distortions caused by FA and SA is our measure of interest:

27 $$\Delta \delta = \delta_{FA} - \delta_{SA,\alpha }$$

Under SA a distinction has to be made between $\upalpha _{SA}^{*}$ and $\uprho _{SA}^{*}$ with respect to the distortional effects. For reasons of avoiding redundancies, we solely focus in the following analysis on the decision variable $\upalpha _{SA}^{*}$. However, we know from the previous analysis that the results for $\upalpha _{SA}^{*}$ and $\uprho _{SA}^{*}$ in relation to the input factors $inv$ and $hr$ are systematically similar in their effects. Thus, it is reasonable to showcase only one decision variable for SA, i.e. $\upalpha$ ($\delta_{SA,\alpha }$).

Both tax systems always distort in the same direction. In case of $\Delta \tau^{ - }$ holds $\alpha_{FA}^{*} < a$ and $\alpha_{SA}^{*} < a,$ thus $\delta_{FA}$ and $\delta_{SA,\alpha }$ are positive. Consequently, a positive [negative] outcome for $\Delta \delta$ (see Eq. (27)) means that the distortions under SA [FA] are lower than those under FA [SA] for $\Delta \tau^{ - } .$

Based on limit considerations, we find for the non-optimality costs $c_{d}$ that the distortion difference $\Delta \delta$ approaches zero in the case where the non-optimality costs $c_{d}$ go toward infinity $(\mathop {\lim }\limits_{{c_{d} \to \infty }} \Delta \delta \to 0)$ as tax considerations become relative to the optimal pre-tax investment decision $a$ negligible. In a case where the non-optimality costs $c_{d}$ go to zero, the optimal decision for tax purposes drives the optimal final investment decisions. From a pure tax-perspective, the entrepreneur would, for $\Delta \tau^{ - }$, under both systems invest all her funds in the low-tax country B. Thus, the difference $\Delta \delta$ approaches zero in this case as well $(\mathop {\lim }\limits_{{c_{d} \to 0}} \Delta \delta \to 0)$ even though the distortions due to taxation are maximal under both systems.

The effects of the variable $tp$ determining the profit shifting possibilities under SA are of highest interest for us, as they i) constitute the most important mechanism influencing the locational decision of the input factors by interacting with the tax rate differential under SA and ii) represent the direct counterpart for real activity shifting under FA. The effects of $tp$ on the difference $\Delta \delta$ are complex and depend on combinations of parameters. However, we can determine straightforwardly that transfer pricing parameter $tp$ for which the distortions caused under both tax base allocation systems are equal (for $tp_{neutr}$ holds $\Delta \delta = 0$).

By equating Eq. (24) and the first part of Eq. (17) and solving for $tp$, we obtain that transfer pricing parameter $tp_{neutr}$ that equalizes distortions under both systems:

28 $$tp_{neutr} = \frac{{c_{G} \left( {\tau_{FA} - 1} \right)\left( {3c_{d} \left( {\tau_{FA} - 1} \right) + \sqrt 3 } \right)\sqrt {c_{d} } \left( {3c_{d} \left( {\tau_{FA} - 1} \right)^{2} + 2\Delta \tau^{2} \pi_{G} } \right)}}{{3\Delta \tau^{2} inv\left( {c_{G} - 1} \right)}},$$

Hence, the transfer pricing parameter $tp$ is characteristic for the direction and the extent of the distortion difference $\Delta \delta$. In the following we investigate the important interaction between the tax rate differential and the transfer pricing strategy for the comparison of distortions caused under both tax base allocation systems. In order to analyze the impact of transfer pricing and the tax rate differential on the distortion difference $\Delta \delta$, we first shed light on the effects under each tax system separately and then bring both perspectives together (see the propositions). In case there is no tax rate differential ($\Delta \tau = 0$), there are no incentives to distort the optimal pre-tax investment decision under either system. Hence, $\Delta \tau \to 0$ always implies $\Delta \delta \to 0$.

If there is a tax rate differential between both countries ($\Delta \tau \ne 0$) no distortions of locational decisions of input factors are caused by SA if and only if no profits can be shifted via transfer pricing ($tp = 0$). Only if no profits can be shifted cross-border, each entity of the MNE is indifferent between producing itself and obtaining products from the other entity. The higher the shiftable profits (i.e. the higher $tp$), the higher the incentives to produce in the low-tax country, sell products to the entity in the high-tax country and thereby shift profits from the high-tax to the low-tax country. Thus, under SA the biggest distortions of the optimal pre-tax investment decisions are caused for maximal profit shifting possibilities ($tp = 1$).

The higher the tax rate differential, the higher are the incentives to deviate from the pre-tax optimum to obtain a tax-optimal group set up. With a higher tax rate differential, more taxes are saved with every product unit shifted cross-border. For the negative [positive] subrange of tax rate differentials $\Delta \tau^{ - }$ [$\Delta \tau^{ + }$], the optimal pre-tax investment decision is always distorted in a negative [positive] way ($\upalpha _{SA}^{*} < a$,$\delta_{SA,\alpha } > 0$ [$\upalpha _{SA}^{*} > a,\delta_{SA,\alpha } < 0$]) under SA. The influence from the profit shifting possibilities $tp$ on the optimal after-tax investment decision $\upalpha _{SA}^{*}$ are not symmetric with respect to the negative and positive subrange of tax rate differentials $\Delta\uptau ^{ - }$ and $\Delta\uptau ^{ + }$. The reason therefore is the different impact of positive and negative tax rate differentials on the optimal after-tax investment decision $\upalpha _{SA}^{*}$ (note the power of three in the denominator):

29 $$\partial \alpha_{SA}^{*} \left( {\Delta \tau } \right) = - \frac{{\left( {1 - a} \right)inv\;tp\left( {1 - \tau_{A} } \right)\pi_{G} }}{{c_d\left( { - 1 + \Delta \tau - a\Delta \tau + \tau_{A} } \right)^{3} c_{G} }}.$$

Consequently, for positive tax rate differentials $\Delta \tau^{ + }$ a marginal increase in the tax rate differential results into a stronger increase in $\alpha_{SA}^{*}$ than for negative tax rate differentials $\Delta\uptau ^{ - }$.

In summary, the highest distortions occur under SA for high tax rate differentials $\Delta \tau$ (even higher for positive tax rate differentials $\Delta \tau^{ + }$ than for negative ones $\Delta \tau^{ - }$) and high profit shifting possibilities $tp$.

Under FA, transfer prices do not play a role. Higher tax rate differentials, however, do also incentivize the entrepreneur under FA to allocate factors in a tax-favorable way. Thus, for higher tax rate differentials the distortions caused under FA are higher as well. Also under FA, a marginal increase in the tax rate differential $\Delta \tau$ results into a stronger increase in $\alpha_{FA}^{*}$ for positive tax rate differentials $\Delta \tau^{ + }$ than for negative tax rate differentials $\Delta \tau^{ - }$. We abandon to show the effects here via formula as it is quite complicated and the proof is provided by the propositions below anyway.

In order to derive conclusions on how the distortion difference develops over different tax rate differentials for different profit shifting possibilities under SA, we combine the two previously described perspectives. Our analysis has proven, that the transfer price is characteristic for the direction and the extent of the distortion difference $\Delta \delta$. To derive general relationships between the distortion difference $\Delta \delta$ and the tax-rate differential $\Delta \tau$ we focus on the partial derivative $\partial \Delta \delta \left( {\Delta \tau } \right)$, which is

30 $$\partial \Delta \delta \left( {\Delta \tau } \right) = \underbrace {{\frac{{\pi_{G} \;inv\;tp\left( {1 - \tau_{A} } \right)}}{{2c_{d} c_{G} \left[ {\tau_{FA} - 1} \right]^{2} }}}}_{part\;I} + \underbrace {{\frac{{\left( {\tau_{A} - 1} \right)\left( {\sqrt 3 c_{d} \left[ {\tau_{FA} - 1} \right] + \sqrt {c_{d} \left( {3c_{d} \left[ {\tau_{FA} - 1} \right]^{2} + 2\Delta \tau^{2} \pi_{G} } \right)} } \right)}}{{2\Delta \tau^{2} \sqrt {c_{d} \left( {3c_{d} \left[ {\tau_{FA} - 1} \right]^{2} + 2\Delta \tau^{2} \pi_{G} } \right)} }}}}_{part\;II}$$

$$with\;\tau_{FA} = \left( {1 - a} \right)\Delta \tau + \tau_{A}$$

We assume $\tau_{A} \wedge \tau_{B} \in \left[ {0,1} \right]$ and $\Delta \tau = \tau_{B} - \tau_{A}$. This implies $\Delta \tau \in \left[ { - 1,1} \right]$ and $\Delta \tau \in \left[ { - \tau_{A} ,1 - \tau_{A} } \right]$. Given this set of assumptions, we state the following propositions:

#### Proposition 1:

*For* $tp < tp_{low}$, $\Delta \delta$ *decreases monotonically over* $\Delta \tau \in \left[ { - \tau_{A} ,1 - \tau_{A} } \right]$.

#### Proposition 2:

*For* $tp > tp_{high}$, $\Delta \delta$ *increases monotonically over* $\Delta \tau \in \left[ { - \tau_{A} ,1 - \tau_{A} } \right]$.

#### Proposition 3:

*For* $tp \in \left[ {tp_{low} ,tp_{high} } \right]$ *two extrema exist. If *$tp \in \left[ {tp_{low} ,tp_{middle} } \right]$, *then solely a minimum over* $\Delta \tau \in \left[ {0,1 - \tau_{A} } \right]$ *exists*. If $tp \in \left[ {tp_{middle} ,tp_{high} } \right]$, *then one maximum over* $\Delta \tau \in \left[ { - \tau_{A} ,0} \right]$ *and one minimum over* $\Delta \tau \in \left[ {0,1 - \tau_{A} } \right]$ *exists*.

From an economic perspective these results show that the distortional effects of FA in comparison to those of SA crucially depend on the possibilities for profit shifting via transfer pricing under SA. We consider the result as paradoxical as for $\Delta \tau^{ + }$ distortions under SA are more severe with high profit shifting possibilities than those under FA. So, even though profits can be shifted simply in the books via transfer pricing, SA may result nonetheless in strong distortions. Reason is that generous profit shifting possibilities incentivize the entrepreneur to invest a big share of funds in the low-tax country in order to use the profit shifting potential maximally. Thus, transfer pricing opportunities and the distortion of the factor allocation have rather a mutually reinforcing effect than—in contrast to commonly held views—a substituting effect. For transfer-prices within $\left[ {tp_{low} ,tp_{high} } \right]$ the different incentives for deviating from the pre-tax optimum may outweigh each other for both tax base allocation systems depending on the tax rate differential. Hence, distortional effects of both systems are ambiguous within this range.

### Illustration of results

**Fig. 2:**
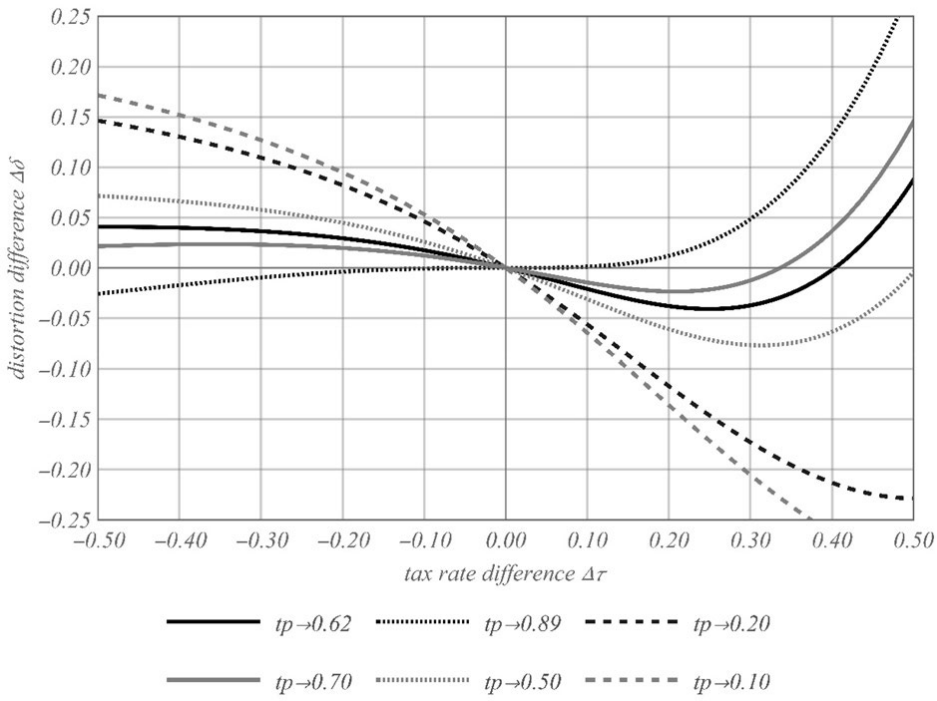
Distortion difference depending on $\Delta \tau$

The following Fig. 2 illustrate our results. It shows the distortional difference depending on the interaction of profit shifting possibilities via transfer pricing under SA and the tax rate differentials. We assume the following parameter settings:

$$\left\{ {hr = 0.5,inv = 0.3,a = 0.5,\tau_{A} = 0.5,c_{d} = 0.1} \right\}$$_._

For this set of parameters, it holds that $tp_{middle} = 0.62$, $tp_{high} = 0.89$ and $tp_{low} = 0.20$. For a positive tax rate differential of $\Delta \tau = 50\%$ (highest label of the x-axis) it holds that $\tau_{B} = 1$; for a negative tax rate differential of $\Delta \tau = - 50\%$ (lowest label of the x-axis) it holds that $\tau_{B} = 0$. Remember that for this illustration $\tau_{A} = 0.5$. For a negative value of the distortion difference $\Delta \delta$ the absolute distortion under FA is bigger than that under SA (note that over $\Delta \tau \in \left[ {0,1 - \tau_{A} } \right]$ the signs of $\delta_{FA}$ and $\delta_{SA,\alpha }$ are negative). There are no distortions under either system if the tax rate differential is zero $(\Delta \tau = 0)$.

The propositions above prove that the amount of $tp$ in combination with the tax rate differential are highly important for the comparison of distortional effects of both systems. Whereas under both tax base allocation systems, tax planning is done via the MNE’s factor allocation between countries, transfer pricing is an additional tool applicable only under SA. From Prop. 1 and 2 it follows that for very bad or good profit shifting possibilities under SA (i.e. lower or higher values of $tp$ than $tp_{low}$ or $tp_{high}$) SA or FA respectively results in bigger distortions independent of the magnitude of the tax rate differential. In line with the reasoning in Prop. 1, the curve for $tp_{low}$ is monotonically decreasing and has its minimum exactly at $\Delta \tau = 50\%$. In contrast and in line with Prop. 2, the curve for $tp_{high}$ is monotonically increasing and its derivative is zero at $\Delta \tau = 0\%$. The distortions for $tp_{high}$ created by SA are, for any tax rate differential in the range of $\Delta \tau \in \left\{ { - \tau_{A} ,1 - \tau_{A} } \right\}$, higher in absolute terms than those created by FA.

For more moderate transfer pricing possibilities (i.e. $tp \in \left[ {tp_{low} ,tp_{high} } \right]$) the magnitude of the tax rate differential becomes decisive for the ranking of the distortive power for both systems over the tax rate differentials. The curves for moderate transfer pricing possibilities cross the x-axis twice: once at $\Delta \tau = 0$ and once more in the range of positive tax rate differentials $\Delta \tau^{ + }$. According to Prop. 3, only one extremum (it is always a minimum) occurs for the distortion difference $\Delta \delta$ for positive tax rate differentials $\Delta \tau^{ + }$ and lower moderate values of $tp$ (i.e. $tp \in \left[ {tp_{low} ,tp_{middle} } \right]$; see the curve for $tp = 0.50$). A minimum only occurs in cases where the curves for the optimal investment decisions under SA and FA intersect in the range of positive tax rate differentials $\Delta \tau^{ + }$. For lower moderate levels of $tp$, the distortion under SA increases from a certain (positive) tax rate differential $\Delta \tau^{ + }$ on upwards stronger than the distortions under FA.

According to Prop. 3 two extrema exist for higher moderate values of $tp$ (i.e. $tp \in \left[ {tp_{middle} ,tp_{high} } \right]$; see the curve for $tp = 0.70$). In this case, the same reasoning holds for the minimum in the range of positive tax rate differentials $\Delta \tau^{ + }$ than that for lower moderate tax rate differentials (see directly above). Additionally, for higher moderate values of $tp$ a maximum occurs for negative tax rate differentials $\Delta \tau^{ - }$. As curves for higher moderate levels of $tp$ do not cross the x-axis for negative tax rate differentials $\Delta \tau^{ - }$ and thus no change of sign in the distortion difference $\Delta \delta$ is caused, the distortions under FA are always stronger for higher moderate levels of $tp$ and negative tax rate differentials $\Delta \tau^{ - }$.

In summary, very good possibilities for shifting profits (high *tp*) via transfer pricing under SA lead in tendency to higher distortions under SA, whereas lower profits shifted by transfer pricing (low *tp*) lead in tendency to higher distortions under FA. For moderate profit shifting possibilities under SA, the distortions are—in tendency—higher under SA if the tax rate differential is positive and high. Otherwise the distortions are bigger under FA for moderate possibilities to shift profits.

For illustrative reasons the reference tax rate $\tau_{A} = 0.5$ is chosen quite high in the graphic. Such a high tax rate is not applied by any EU country. However, for lower levels of $c_{d}$ than that used for creating the graphic (i.e. $c_{d} \to 0.1$) the intersection for moderate transfer prices.

($tp \in \left[ {tp_{low} ,tp_{middle} } \right]$) with the x-axis in the positive range for tax rate differential $\Delta \tau^{ + }$ happens for lower level of taxes and for lower tax rate differentials. Thus, the switch in the ranking for the tax base allocation system that distorts more severely is not just a theoretical case but might be frequently observable for many European country pairs. As the nominal tax rates vary quite strongly in EU countries and as countries on average change their tax rates rather frequently (in tendency they decrease them) a small change in the tax rate differential might result in in a changed rank order of the distortive power of both tax base allocation systems for a given country pair.

## Discussion

In contrast to claims of the European Commission, our analysis shows that a shift from SA to FA does not necessarily result in a system that leads to less severe distortions. According to the official justification of the latest proposal, “the CCCTB falls within the ambit of the Commission’s initiatives for fairer taxation and would contribute to the elimination of obstacles which create distortions that impede the proper functioning of the internal market” (European Commission 2016a). At least with respect to locational investment decisions, it is far from clear whether distortions are mitigated compared to the current situation under SA. In order to be able to assess the consequences of such a switch in systems, information about actual transfer pricing possibilities (i.e. the $tp$ in our analysis) has to be (publicly) available. As this information is not widely available for policy makers and tax researchers, the economic consequences from introducing a CCCTB are not foreseeable. In so far, the behavior of the European Countries hesitating to introduce a CCCTB with the elements of consolidation and formula apportionment seems to be reasonable from our perspective.

We outline that anticipating effects of the introduction of a CCCTB with missing information on transfer pricing parameters is misleading. However, if the missing information becomes available, our study provides strong support for anticipating potential behavioral consequences of firms and thus for fiscal revenues. Thus, our results contribute to supporting the decision process of the Member States for the implementation of a Europe-wide tax system based on FA.

Under SA, only transfer prices valued at costs would not result in distortions of the factor allocation between countries. However, transfer prices valued at costs contradict the arm’s-length principle and are not a practicable solution to tackling distortional effects caused by SA. The more profits can be shifted via transfer pricing, the higher are the distortions that are created by SA. Thus, in line with the results of Martini et al. (2012), yet in contrast to some commonly held views andJuranek et al. (2018),^14^ we find that the application of transfer pricing under SA has real economic effects. Transfer pricing opportunities and the distortion of the factor allocation under SA have rather a mutually reinforcing effect than a substituting effect.

Our results show that the introduction of FA has no clear effect on the magnitude of distortions. Even though applied transfer prices are not publicly available, we can, at least to some extent, derive conclusions about distortional effects created by each tax system for particular industries. Companies engaging in traditional industries might have fewer opportunities to shift profits via transfer pricing, as they are physically present in each country they do business in and as their products are tangible. Thus, tax authorities can easily inspect the product flows within the company. Moreover, there are more likely standardized benchmark prices for the products they sell. In contrast, transfer prices for intellectual property and intangible assets are particularly difficult to be priced, as usually no benchmarks exist. Thus, profits can be shifted more easily for e.g. companies of the digital industry whose business model is largely based on IP and intangible assets in the form of data (see Stöwhase 2002). Assuming very low [high] profit shifting potential for the traditional [digital] industry our findings indicate that the traditional industry reacts with more heavily distorted factor allocations between countries on a shift from SA to FA, whereas the digital industry adjusts its factor allocation to a more moderate (less distorted) level after the shift.

Furthermore, our results indicate that the adaptations in the factor allocation between countries are stronger for high tax rate differentials for a shift from SA to FA. The corporate tax rates for Europe (see KPMG 2019) show that in the most extreme cases, nominal tax rates differentials are quite high and amount up to 26 percentage points (nominal tax rate in Hungary 9%, Malta 35%). The possibility of sharing digital content is basically limitless across borders and the unconditional availability of information around the globe likely decreases the “non-optimality costs” $c_{d}$ for the optimal company set-up from a pre-tax perspective. Thus, the driving force tightening up the pre-tax with the after-tax investment decision is less strong for companies of the digital economy. Consequently, those companies are more strongly incentivized to set up a tax-favorable firm structure under both tax systems. Plenty of real-life cases, like the incorporation of lots of digital companies in Ireland, might support this result. Due to the higher flexibility in the business structure of digital companies, they are more likely to adjust their structure in a very tax-favorable way under SA and cherry-pick a low-tax country as a location for one entity. Consequently, the tax rate differential between the countries of domicile of the MNE are likely to be characterized by high tax rate differentials. Thus, even stronger adaptation reactions of digital MNEs need to be expected in case of a shift from SA to FA. In contrast, real economy entities are more dependent on the resources in a country and have more limited opportunities for cherry-picking low-tax countries as countries of domicile for the MNE. Thus, the tax rate differentials between countries of residence of these types of MNEs might be lower in tendency. Thus, the adaptation reactions of them are, with respect to tax rate differentials, expected to be less strong for a shift from SA to FA.

Due to consolidation and an apportionment formula based on equally weighted factor weights, the optimal decisions for investments in assets $$inv$$ and workforce $$hr$$ are always equal under FA ($$\alpha_{FA}^{*} =$$$$\rho_{FA}^{*}$$). The absolute magnitudes of assets $$inv$$ and workforce $$hr$$ have no impact on this outcome (as long as the overall investment is positive, i.e. $$\pi > 0$$). Under FA only the allocation between the countries counts. The relative magnitude of one apportionment factor to the magnitude of the other factors does not matter under FA as the factor weights are fixed. In contrast, under SA, the two after-tax investment decisions ($$\alpha_{SA}^{*}$$,$$\rho_{SA}^{*}$$) are highly dependent on the magnitudes of investment in assets $$inv$$ and workforce $$hr$$. Only in case assets $$inv$$ equal workforce $$hr$$ holds $$\alpha_{SA}^{*} =$$$$\rho_{SA}^{*}$$(provided that $$\Delta \tau \ne 0$$). Thus, a switch from SA to FA would incentivize MNEs to equally distribute assets and workforce between countries (in relative terms not in absolute ones). We do not focus on sales here as they are not, or are at least less manipulatable and cannot be planned well by MNEs.

We focus in this model solely on income shifting via transfer pricing under SA. We know from the literature (Nicolay et al. (2017), Schindler and Schjelderup (2016)) that also internal debt shifting is a very powerful tool for income shifting under SA. Under SA, internal debt shifting results in relocating capital costs and not only pure profits. However, if we suppose the rules of the Council Directive of a CCCTB (2011) for the tax treatment of interest payments and financial assets under FA, income shifting via internal debt is eliminated under FA (see Schreiber 2009). Interest payments and interest income for granting loans between group members are consolidated and thus, do not affect the taxable profits. Moreover, interest income is by definition not included for calculating the allocation factor “sales” for regular, non-specific companies. Additionally, financial assets do neither affect the allocation factor “assets”. Thus, against the background of our paper, the alternative income shifting channel internal debt takes only effect under SA. Schindler and Schjelderup (2016) state that internal debt shifting and transfer pricing are cost substitutes. Thus, the effects of transfer pricing under SA might in tendency be overstated in our model. The only tax effect of external debt under FA is the reduction of the consolidated group tax base. In contrast to SA, there is no possibility to assign tax-favorable interest expenses to a certain entity, which e.g. is located in a high-tax country. Consequently, also external debt can be used in a way more tax-beneficial way under SA than under FA.

## Conclusion

We examine which tax base allocation system leads to more severe distortions with respect to locational decisions of the input factors assets and workforce. While existing studies focus primarily on the impact of taxes on locational decisions under either separate accounting (SA) or formula apportionment (FA), the main innovation of this paper is that it compares both systems with regard to the level of distortions they induce and identifies the conditions under which distortions are high or low. We model after-tax investment decisions of a centrally managed MNE under both tax allocation systems, whereas the optimal pre-tax investment decision is given by relative demand of each country. The optimal pre-tax investment decision serves as a benchmark for the level of distortion caused by each system. Whereas under FA tax planning can only be done via the MNE’s allocation of factors (real activity shifting) under SA shifting book profits via transfer pricing is an additional tool for tax planning. We abstract in this paper from the alternative profit shifting channel of internal debt.

We make the following contributions to the literature. First, in contrast to claims of the European Commission, our analysis shows that a shift from SA to FA does not necessarily result in less severe distortions. At least with respect to locational investment decisions, it is far from clear whether distortions are mitigated compared to the current situation under SA. In order to be able to assess the consequences of such a switch in systems, information about actual transfer pricing possibilities (i.e. the $tp$ in our analysis) has to be (publicly) available. Even though researchers investigated profit shifting behavior of MNEs intensively and can e.g. deduce adaptations in profit shifting to changed tax rates (Alexander et al. 2020; Delis et al. 2020; Lohse & Riedel 2013), we still have almost no information on applied transfer prices and on the average share of sales or profits that is shifted via transfer pricing. As this detailed transfer pricing information is not generally available, not even the direction of the distortional effects caused by a switch in systems is anticipatable. Our results contribute to supporting the decision process of the Member States for a Europe-wide tax system as we outline the importance of becoming better aware of the status quo. We show that the introduction of FA has no clear effect on the direction and the magnitude of distortions. For companies that, in tendency, shifted only minimal profits via transfer pricing, a shift from SA to FA would incentivize them to adjust their business structure in a way that creates more severe distortions with respect to locational decisions. In contrast, firms that were able to shift a lot of profits via transfer pricing under SA would be forced to set-up a more moderate and less distorted business structure. A potential example for an industry that might be able to shift a lot of profits via transfer pricing under SA is the digital industry. In contrast, profit shifting potentials for the traditional industry might be rather low under SA. Thus, the switch from FA to SA would reduce distortions in the digital industry but increase them for the traditional industry.

Second, in line with the results of Martini et al. (2012), yet in contrast to some commonly held views,^15^ we find that the application of transfer pricing under SA has real economic effects. This result is to some extent paradoxical. Even though profits can be shifted simply in the books via transfer pricing, SA causes nonetheless strong distortions as high profit shifting possibilities incentivize the entrepreneur to invest a big share of funds in the low-tax country in order to use the profit shifting potential maximally. Thus, transfer pricing opportunities and the distortion of the factor allocation have rather a mutually reinforcing effect than a substituting effect. Moreover, in contrast to the often-stated insensitivity of FA toward income shifting, we prove that the introduction of a tax allocation system based on FA in Europe could lead to a severe shift of economic substance to low-tax countries. The reason for this is that under FA, the only mean for tax planning is factor allocation, while under SA, tax planning is a mixture of book profit shifting via transfer pricing and factor allocation.

Finally, under FA the optimal after-tax investment decisions for the input factors assets and workforce are always similar in relative terms, whereas under SA they differ if investments into assets and workforce differ. Thus, a shift from FA to SA would likely result in a more equal allocation of assets and workforce between countries. Moreover, we show that very high non-optimality costs, as might be typical for the traditional industry, result in low distortional effects for both tax systems as any kind of tax considerations become negligible. Thus, for very high non-optimality costs, the magnitude in distortions created by both systems is equal, i.e. close to zero. However, also for very low non-optimality costs, the magnitude in distortions created by both systems is equal even though the level of distortion is maximally high under both systems. As both systems distort in the same direction, the MNE invests in a very tax-favorable way under both systems, meaning they invest all funds in the low-tax country. A typical industry with low non-optimality costs might be the digital industry.

The results of this paper are of particular interest for European and OECD policy-makers. On an aggregated level, our results help anticipating the macroeconomic effects induced by the (potential) introduction of a tax base allocation system based on FA, e.g. against the background of the CCCTB system or Pillar One of the global tax reform. As we take a business perspective, our results may induce MNEs to reassess their recent locational investment decisions in the face of a potential future change in the applied tax base allocation system.

From our results we can derive some important policy implications. Without knowing the status quo with respect to the share in sales or profits shifted via transfer pricing under the current SA system, it is impossible to assess the consequences for distortional effects of locational decisions. When the status quo is known, our results help anticipate MNEs’ adjustments of the company structure.

Our findings—as always in analytical research—must be interpreted against the background of our set of assumptions. Our results are, to some extent, driven by the highly stylized setting we apply. The application of non-optimality costs accounts in our model for the inefficient usage of input factors. This is a heuristic that might be challenged, e.g. if the output quantity *Y* is not reasonable high in relation to factor costs ${{c_{1} } \mathord{\left/ {\vphantom {{c_{1} } {c_{2} }}} \right. \kern-0pt} {c_{2} }}$. Our analysis does not include adaptations of investment volumes or production quantity if taxation is introduced or the applied system is switched from SA to FA. We assume a two-country setting. Results for an MNE located in more countries might be qualitatively the same, but the effects are likely less strong. Moreover, assuming the same productivity for assets and workforce might not be realistic for all industries. Several important issues have not yet been sufficiently addressed. Hence, our study contributes to answer relevant, but not, by far, all questions concerning the different distortional effects of SA and FA.

### Supplementary Information

Below is the link to the electronic supplementary material.

Supplementary file1 (DOCX 73 KB)

## Data Availability

For this research project no data was generated or analyzed.

## Footnotes

1 See Martini et al. (2012).

2 Baker & English (2011), p. 97.

3 We focus here on high-skilled workers as they usually add the biggest share of value to products. Empirical evidence shows that high-skilled workers remain complementary to machines, rather than being replaced by them (see Alasoini and Tuomivaara 2022 and Hawksworth et al. 2018).

4 As the demand in the two considered countries is exactly met by the production output of the MNE and the market size does not change, the (re-)investment opportunity is only given once and cannot be mimeographed. We assume that the investment does not affect market prices for production factors and goods sold. The investment budget ${c}_{G}$ is exactly required to generate the sales $S=1$, meaning that an investment budget below ${c}_{G}$ would not result in sales $S=1$. The investment budget ${c}_{G}$ is assumed to be optimal.

5 Even though the production conditions are similar in both countries, the difference in tax rates might in line with the results of Haufler and Wootton (1999) be reasoned by different market sizes.

6 Devereux & Griffith (1998), p. 336 focus on transportation costs as the crucial factor in deciding where to produce. Whereas we borrow their general line of argumentation for justifying a pre-tax optimum by cost-reasons, we do not simply focus on transportation costs but take a more general concept of non-optimality costs into account.

7 Also assumed by Oestreicher & Koch (2011).

8 Note that the application of the profit split method based on investment per country is in the context of our model set-up nothing else than formula apportionment based on the factors workforce and assets. Thus, if this specific transfer pricing method is applied under SA, tax effects are identical to those under FA. However, the German fiscal authorities generally still hesitate to apply this method as they prefer applying the standard transfer pricing methods. Thus, in practice, the use of standard transfer pricing methods as we capture them in our model is widespread (see Hanken et al. 2020).

9 Martini et al. (2012) assume given transfer prices as well.

10 Nielsen et al. (2010) assume costs for transfer pricing as they are not ruling out that the MNE only engages in transfer pricing in line with the concepts of the tax authorities.

11 Only in the calculation of the factor “labor” does the proposed formula under the CCCTB system deviate slightly from the Massachusetts Formula. Whereas the labor factor consists only of the expenses for the workforce under the Massachusetts Formula, it consists in equal parts of those expenses and number of workers under the proposed CCCTB formula. However, this small simplification of the Massachusetts Formula is negligible for the purpose of our analysis.

12 Even though there might be room for manipulating the allocation of assets and workforce on a book basis (e.g. manipulate assets by leasing arrangements) we believe the effect is by far not as strong as the one for book profit shifting created by transfer pricing under SA. Thus, we abstract from these possibilities under FA (for an investigation see Kiesewetter et al. 2018).

13 As Eq. (12) is a quadratic function, setting $\partial \pi_{G}^{FA} \left( \alpha \right)$ equal to 0 and solving for $\alpha$ results into two potential solutions. However, only the solution shown in Eq. (13) leads to a maximum.

14 They find that under the traditional transfer pricing methods mispricing of royalty payments does not affect investment behavior. However, transfer pricing in royalties is a rather special case.

15 See Martini et al. (2012).
