# Supplementary material for: Distortional effects of separate accounting and formula apportionment on factor allocation
Source: J Bus Econ. 2023 Jan 6;93(8):1277–307. doi: 10.1007/s11573-022-01133-5 (PMC10515356; doi:10.1007/s11573-022-01133-5)
Supplement: Supplementary file 1 — Supplementary file1 (DOCX 73 KB) [file 11573_2022_1133_MOESM1_ESM.docx]

# 6 Appendix

**Proof of Proposition 1:** $\Delta\delta$ decreases, if $\partial\Delta\delta\left(\Delta\tau\right)<0$ over $\Delta\tau\in\left[-\tau_{A},1-\tau_{A}\right]$ holds. The derivative $\partial\Delta\delta\left(\Delta\tau\right)$ is a sum consisting of two parts. Part I is positive infinite at $\Delta\tau\to\frac{\tau_{A}-1}{a-1}$. Hence, for $\Delta\tau<\frac{\tau_{A}-1}{a-1}$ it is strictly convex. Part I increases linearly with *tp*. With increasing *tp* convexity of part I decreases.

The denominator of part II may have three roots. One is $\Delta\tau\to0$, the other two are

. $\lim_{\Delta\tau\to0} \text{part} \text{II<0}$. Denote, that part II is no function of *tp*. Therefore, for $tp\to0$ $\partial\Delta\delta\left(\Delta\tau\right)$ is non-positive for all $\Delta\tau$. That rules out an extremum. As part I is convex for $\Delta\tau<\frac{\tau_{A}-1}{a-1}$ and increases with *tp*, for specific transfer price $tp_{low}$ $\lim_{\Delta\tau\to1-\tau_{A}} \partial\Delta\delta\left(\Delta\tau\right)=0$ must hold:

$tp_{low}=\frac{a^{2}c_{d}c_{G}\left(1-\sqrt{3}a\sqrt{\frac{c_{d}}{3a^{2}c_{d}+2\pi_{G}}}\right)}{\pi_{G} inv}$.

Due to decreasing convexity of part I with increasing *tp*, $tp<tp_{low}$ rules out any extremum over $\Delta\tau\in\left[-\tau_{A},1-\tau_{A}\right]$ ■.

**Proof of Proposition 2:** An extremum requires $\partial\Delta\delta\left(\Delta\tau\right)=0 \text{and }\partial^{2}\Delta\delta\left(\Delta\tau\right)\neq0$. As part I and part II are different functions of $\Delta\tau$, $\partial\Delta\delta\left(\Delta\tau\right)=0 \text{and }\partial^{2}\Delta\delta\left(\Delta\tau\right)\neq0$ may only occur at $\Delta\tau\to0$. For $tp_{high}\to\frac{c_{G}}{3 inv}$ $\partial\Delta\delta\left(\Delta\tau\right)=0 \text{and }\partial^{2}\Delta\delta\left(\Delta\tau\right)=0$ holds. As part I is strictly convex over $\Delta\tau\in\left[-\tau_{A},1-\tau_{A}\right]$ for $tp>tp_{high}$ no extremum exists ■.

**Proof of Proposition 3:** Part I is strictly convex over $\Delta\tau\in\left[-\tau_{A},1-\tau_{A}\right]$, part II is not. For $tp>tp_{low}$ $\lim_{\Delta\tau\to1-\tau_{A}} \partial\Delta\delta\left(\Delta\tau\right)>0$. For any $tp<tp_{high}$ $\lim_{\Delta\tau\to0} \partial\Delta\delta\left(\Delta\tau\right)<0$. Therefore, a minimum over $\Delta\tau\in\left[0,1-\tau_{A}\right]$ exists. If $\lim_{\Delta\tau\to-\tau_{A}} \partial\Delta\delta\left(\Delta\tau\right)>0$, then a maximum over $\Delta\tau\in\left[-\tau_{A},0\right]$ exists. $\lim_{\Delta\tau\to-\tau_{A}} \partial\Delta\delta\left(\Delta\tau\right)=0$ holds for

$tp_{middle}=\frac{c_{d}c_{G}\left(a \tau_{A}-1\right)^{2}\left(1+\frac{\sqrt{3}c_{d}\left(a\tau_{A}-1\right)}{\sqrt{c_{d}\left(3c_{d}\left(a \tau_{A}-1\right)^{2}+2\pi_{G}\tau_{A}^{2}\right)}}\right)}{\pi_{G} inv \tau_{A}^{2}}$**.**

Part I is strictly convex. Hence, for $tp_{low}<tp<tp_{middle}$ no maximum over $\Delta\tau\in\left[-\tau_{A},0\right]$ exists ■.
